# Editorial: Recognizing Microexpression: An Interdisciplinary Perspective

**DOI:** 10.3389/fpsyg.2019.01318

**Published:** 2019-06-04

**Authors:** Xunbing Shen, Wenfeng Chen, Guoying Zhao, Ping Hu

**Affiliations:** ^1^Department of Psychology, Jiangxi University of Traditional Chinese Medicine, Nanchang, China; ^2^Department of Psychology, Renmin University of China, Beijing, China; ^3^Center for Machine Vision and Signal Analysis, University of Oulu, Oulu, Finland

**Keywords:** macro-expression, micro-expression, recognition, computer vision, cognitive neuroscience

As a Chinese saying goes, “Look at the weather when you step out; look at people's faces when you step in.” Recognizing expressions is a very common activity in daily life. People can infer someone's inner emotions from his or her facial expressions. However, human do not always wear her heart on her sleeves; someone may suppress the expressions of true feelings and express a false facial expression depending on the context of cultural rule or his/her intention. The suppressed expressions can be leaked fleetingly in the form of micro-expressions, which usually last for only 1/25 to 1/5 s.

Microexpressions may reveal the genuine inner emotion and feelings, and are important for many practical applications, such as national security, deception detection, clinical therapy, consumer behavior analysis, and human-computer interaction. Microexpression recognition is an interdisciplinary field attracting a large amount of efforts from researchers in psychology, neuroscience, and computer science. This topic illuminates the latest advances in interdisciplinary understanding how microexpressions are perceived and recognized. The authors contribute from diverse perspectives in the current research topic by using behavioral experiment, EEG, fMRI, and computer vision techniques. They investigated how human recognize macroexpressions and microexpressions in term of modulating factors (e.g., gender, duration) and the underlying neural mechanism, and how machine recognition algorithms and models are developed and inspired by the human recognition data.

Gender will influence the recognition of macro-expressions, Liu et al. investigated the interaction between facial expressions and facial gender information during face perception by using EEG technique. They found that the processing of facial expressions could affect the processing of gender in the early and later stages, which indicated by the early (P1) and late (LPC). The results provide some insights for future work on the recognition of micro-expression.

Previous studies (Adolphs, 2002) showed that the perceiver would mimic the observed expressions while recognizing them; there are close relationships between the production of facial expressions of emotion and recognition of them. From the perspective of expression production, Qu et al. investigated the awareness of facial micro-expressions and macro-expressions (all expressions last for less than 4 s). They found awareness rates were 57.79% in the real-time condition and 75.92% in the video-review condition, and the awareness rate was influenced by the intensity and (or) the duration of facial expressions.

Microexpressions is characterized as dynamic, the features of dynamic expressions may be essential for the recognition of microexpressions. Pfister et al. (2011) started pioneering research on spontaneous micro-expression recognition with the first machine vision framework to recognize spontaneous micro-expressions and achieved very promising results that compare favorably with the human accuracy. Their most recent work integrating micro-expression recognition and detection has been also reported by MIT Technology Review (see http://www.technologyreview.com/view/543501/machine-vision-algorithm-learns-to-recognizehidden-facial-expression) and achieved increasing attention (Li et al., 2018). Guo et al. investigated the dynamic features of lip corners and their characteristics in genuine and posed smiles. They found that the genuine smiles have higher amount of onset, apex, offset, and total durations, as well as offset displacement compared to posed smiles; however, the amount of onset and offset speeds, and symmetry tended to be lower. Based on these results, Li et al. regarded the deep learning as a very promising method in the automatic recognition of micro-expression.

Is micro-expression recognition a variant of recognition of macro-expression, or is it a wholly distinctive neurological process? The answer may be the latter (Shen et al., 2016). To further reveal the neural mechanisms underlying the recognition of micro-expressions, Zhao et al. investigated the brain area activities while recognizing micro-expressions of fear and surprise, they found that fear micro-expression recognition evoked greater activities in the left precuneus, middle temporal gyrus, middle frontal gyrus, and right lingual gyrus; the right postcentral gyrus and left posterior insula were responsible for the recognizing surprise micro-expressions.

It is hard for naïve human to recognize micro-expression. Usually, researchers analyze the video clips containing micro-expressions by going through them frame by frame, which is time-consuming and inefficient. To find an effective algorithm for automatically recognizing micro-expression, Peng et al. developed a Dual Temporal Scale Convolutional Neural Network (DTSCNN) for spontaneous micro-expressions recognition. They used two micro-expression databases (CASME I/II, see Yan et al., 2014) to validate the algorithm and the results showed that the method achieved a recognition rate almost 10% higher than what other state-of-the-art methods can achieve.

Automatic facial micro-expression analysis has received increasing attention in the area of computer vision. However, the limitations of current literatures exist, e.g., microexpression database and effective algorithm are fewer. Oh et al. presented a comprehensive review of state-of-the-art databases and methods for micro-expressions recognition, and pointed out the challenges and future directions in the field of automatic facial micro-expression analysis.

During the interpersonal communication, other's facial expressions such as smiling can affect decision making. He et al. investigated the effects of smiling on the responses in ultimatum games, in which they found that smiling of the proposer can lead to a lower average rejection rate.

Together, the topic reveal that research on the recognition of microexpressions are diverse but progressing. This is not surprising given that it receives more and more attention due to the promising potential applications. As new techniques and theories develop, it is likely that efficient and effective algorithms for recognizing microexpression are promising. We hope that these articles provide a look into that future.

## Author Contributions

XS had primary writing responsibility. WC and GZ revised the manuscript. PH assisted with the preparation of the manuscript.

### Conflict of Interest Statement

The authors declare that the research was conducted in the absence of any commercial or financial relationships that could be construed as a potential conflict of interest.
